# Post-Stevens-Johnson Syndrome Bronchiolitis Obliterans: Report of a Complex Case and a Literature Review

**DOI:** 10.7759/cureus.74181

**Published:** 2024-11-21

**Authors:** Ahmed Fadel, Yasser N Ahmed

**Affiliations:** 1 Respiratory Medicine, Dartford and Gravesham National Health Service (NHS) Trust, Dartford, GBR

**Keywords:** air trapping, bronchiolitis obliterans, high-resolution ct (hrct), lung transplantation, mosaicism, ocular stevens-johnson syndrome sequelae, pulmonary complications, small airway disease

## Abstract

Bronchiolitis obliterans (BO) is a rare and severe respiratory complication of Stevens-Johnson syndrome (SJS), which primarily affects the small airways and causes progressive respiratory decline. We present the case of a young male with autism spectrum disorder who developed BO after an episode of SJS triggered by amoxicillin. Initially, the patient presented with an ulcerative rash and respiratory symptoms and received high-dose corticosteroids, leading to partial resolution of mucocutaneous lesions. However, his respiratory symptoms progressively worsened, and he was initially misdiagnosed with asthma, which delayed the appropriate treatment. Imaging and pulmonary function tests (PFTs) revealed findings consistent with BO, including mosaic attenuation, airway thickening, and severe obstruction without reversibility. After infectious and autoimmune causes were ruled out, management included high-dose corticosteroids and fluticasone, azithromycin, and montelukast (FAM) therapy, resulting in partial clinical improvement. Recognising the progressive nature of post-SJS BO, the patient was referred to a tertiary centre for lung transplantation. This case highlights the importance of early diagnosis and tailored management, including potential transplantation, in patients with severe post-SJS BO.

## Introduction

Stevens-Johnson syndrome (SJS) is an uncommon but potentially life-threatening condition characterised by extensive mucocutaneous blistering, typically triggered by medications or infections. Respiratory complications in SJS are rare but can be severe, including the development of bronchiolitis obliterans (BO), an obstructive lung disease affecting the small airways. Diagnosing BO in patients with SJS is challenging, as the initial symptoms often mimic asthma, potentially delaying targeted intervention. Imaging, especially high-resolution computed tomography (HRCT) and pulmonary function tests (PFTs), is crucial for diagnosis, typically showing mosaic attenuation, airway thickening, and air trapping. Although treatment options are limited, fluticasone, azithromycin, and montelukast (FAM) therapy has shown promise in certain cases, whereas lung transplantation may be necessary for patients with progressive disease who are unresponsive to medical management. This case underscores the need for early recognition, comprehensive pulmonary evaluation, and multidisciplinary intervention to improve outcomes in post-SJS BO [1].

## Case presentation

**Figure 1:**
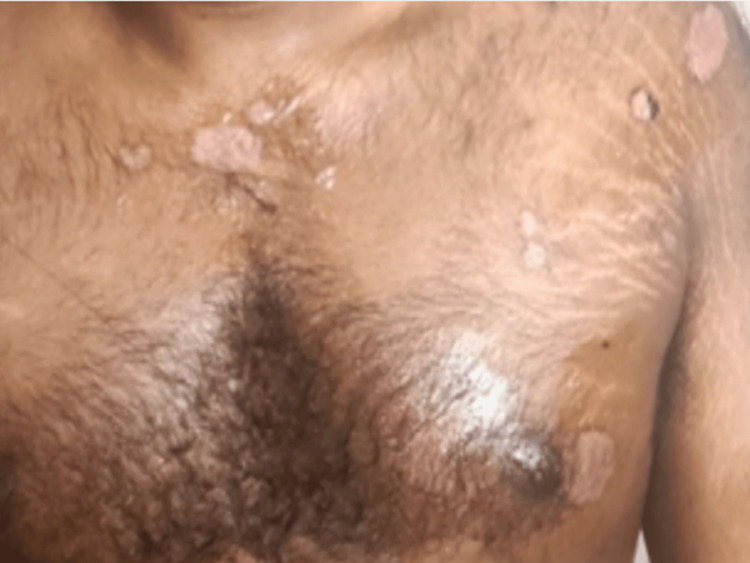
Ulcerative rash less than 10% of the body surface area

A 19-year-old male with autism spectrum disorder, no personal drug history, drug hypersensitivity, no history of smoking, vaping, occupational exposure, no family history of hereditary diseases, and no previous respiratory conditions presented to the emergency department (ED) with an ulcerative rash involving less than 10% of the skin, eyes, and mouth. This rash appeared three days after initiating oral amoxicillin treatment for a presumed upper respiratory tract infection (Figure 1).

**Figure 2:**
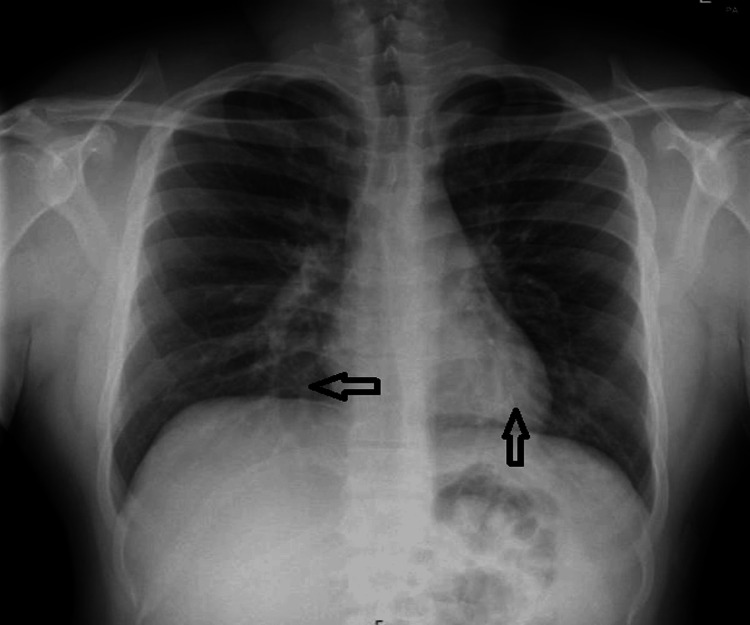
Chest X-ray: bilateral nonspecific inflammatory changes

The patient was diagnosed with SJS and was started on high-dose corticosteroids, which resulted in a gradual improvement of his mucocutaneous lesions. During his hospital stay, he developed a cough and shortness of breath, initially suspected to be due to a chest infection. Chest radiography revealed nonspecific inflammatory changes (Figure 2), and the patient was treated with the non-penicillin antibiotic levofloxacin. The patient was subsequently discharged with a tapered dose of oral prednisolone to continue treatment for SJS.

**Figure 3:**
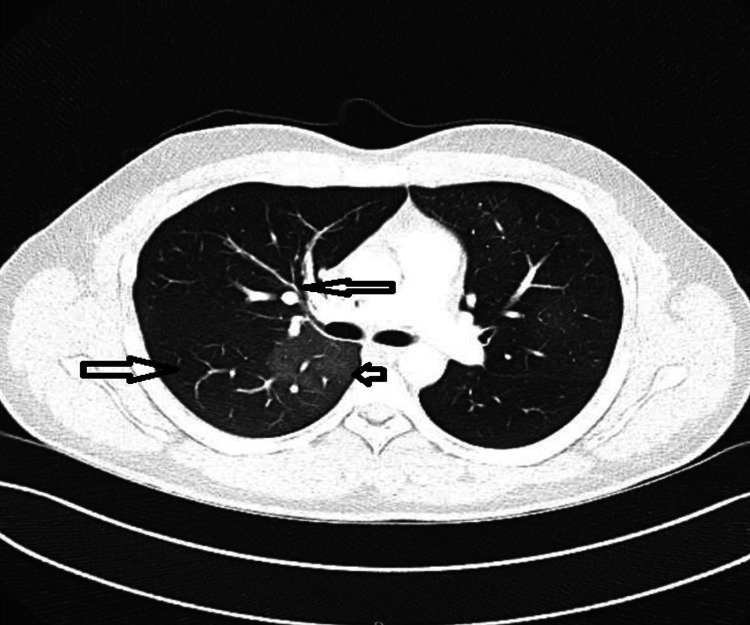
Airway thickening, oligemia, mosaicism (airway trapping)

**Figure 4:**
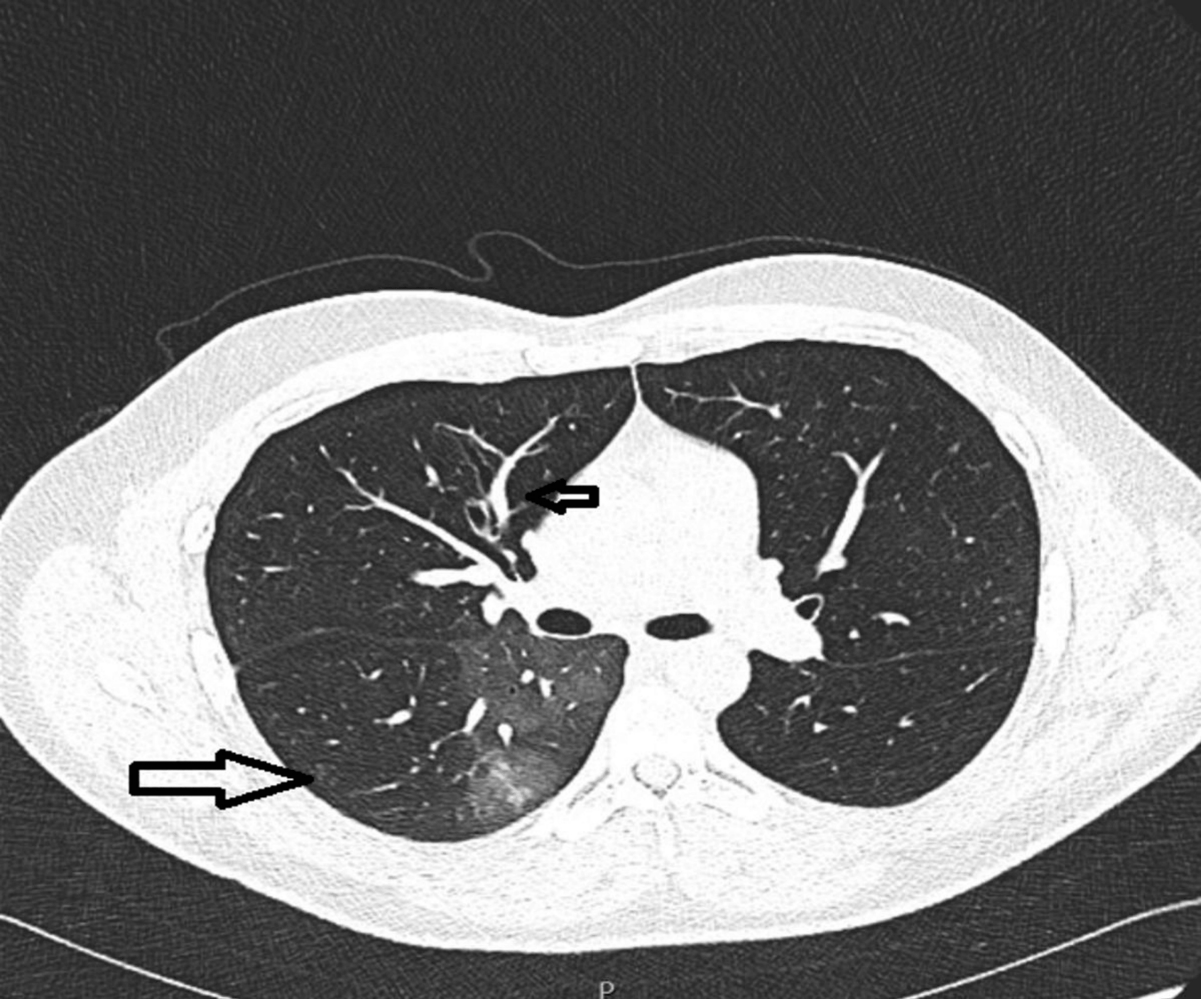
Progression of air trapping, mosaicism, oligemia, vascular attenuation

Two weeks after discharge, the patient returned to the ED with worsening shortness of breath and noisy breath. Initially managed conservatively, he continued to present to the ED almost weekly with progressively worsening respiratory symptoms. Owing to the persistence of his symptoms, CT pulmonary angiography (CTPA) was performed to rule out pulmonary embolism (PE) (Figure 3 and Figure 4). While the CTPA excluded PE, it revealed airway abnormalities, including airway thickening, oligemia, mosaic attenuation, and air trapping - findings indicative of small airway disease. Unfortunately, despite these findings, the patient was discharged with a clinical diagnosis of asthma and prescribed inhalers and montelukast without referral to the respiratory team for further evaluation.

Despite the patient’s continued progressive shortness of breath and multiple visits to the ED, where he was treated for acute asthma exacerbation, his symptoms persisted. Spirometry was performed, revealing severe obstruction with a forced expiratory volume in one second (FEV_1_) of 0.59 L (14% of predicted) without reversibility, forced vital capacity (FVC) of 0.81 L (17% of predicted), and an FEV_1_/FVC ratio of 0.63 (73%). The patient was referred to a respiratory team for further evaluation.

**Figure 5:**
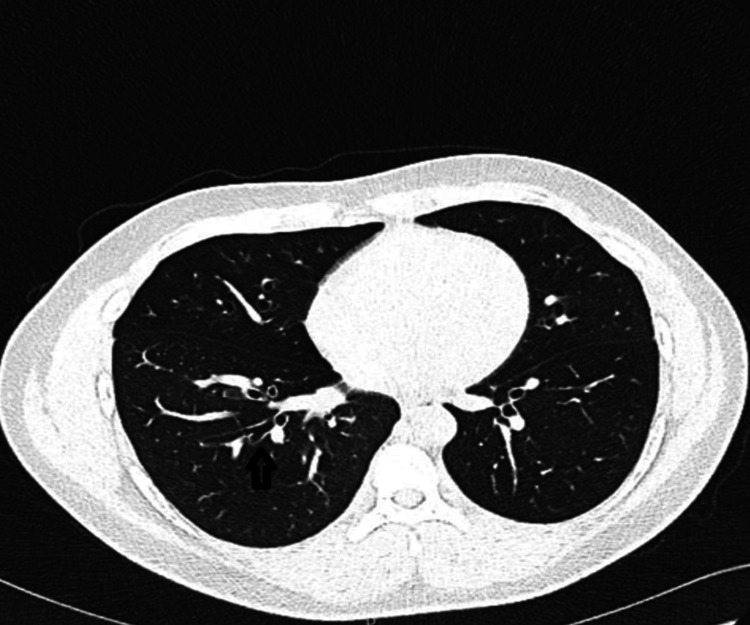
Progression of airway thickening, bronchiectasis

The respiratory team reviewed the patient’s history and CTPA findings, which raised suspicion of BO. A diagnosis of post-SJS BO was considered. To further assess the airway changes, HRCT of the chest was ordered; however, due to significant breathlessness, the patient was unable to complete both the inspiratory and expiratory phases. HRCT showed progression of airway thickening, bronchiectasis, air trapping, mosaic attenuation, oligemia, and vascular attenuation (Figure 4 and Figure 5), further supporting the diagnosis of BO.

An extensive workup, including assessments for infectious and autoimmune causes, was performed (Table 1). This workup effectively excluded both autoimmune and infectious aetiologies.

**Table 1:** Workup done, including infectious and autoimmune causes CCP, cyclic citrullinated peptide; PCR, polymerase chain reaction; RSV, respiratory syncytial virus

Investigation	Result	Units	Normal Reference Level	Interpretation
Immunoglobulin E	166	International units per millilitre (IU/mL)	<100 IU/mL	Elevated
Immunoglobulin E (repeat)	69	IU/mL	<100 IU/mL	Normalised
Antineutrophil cytoplasmic antibodies screen	Negative	-	Negative	Normal
Antinuclear antibody	Negative	Titre	<1:40	Normal
HIV antibody	Negative	-	Negative	Normal
Mycoplasma sputum PCR	Negative	-	Negative	Normal
Pertussis serology	Negative	-	Negative	Normal
Extended viral PCR (including influenza, parainfluenza, RSV)	Negative	-	Negative	Normal
Rheumatoid factor	Negative	IU/mL	<14 IU/mL	Normal
Anti-CCP antibody	Negative	Units per millilitre (U/mL)	<20 U/mL	Normal

Upon establishing the diagnosis, prednisolone was initiated at 1 mg/kg, with a plan for gradual weaning. Additionally, high-dose inhaled corticosteroids, a long-acting beta-agonist (LABA), and a long-acting anti-muscarinic agent (LAMA) were introduced along with montelukast and azithromycin (500 mg three times weekly). Long-term azithromycin therapy alone has shown potential in slowing the progression of BO [2-3].

The patient was discharged on the aforementioned regimen, with follow-up arranged by a respiratory nursing team who visited him at his house. The patient showed slow but steady clinical improvement in breathlessness over time.

A full lung function test was conducted two months after discharge (Table 2), revealing the following findings:

**Table 2:** Lung function test

Test Parameter	Measured Value	Predicted Value	% Predicted	Interpretation
Vital Capacity (VC Max)	1.80 L	4.04-5.88 L	36.2%	Severely reduced
Forced expiratory volume in one second (FEV_1_)	0.58 L	3.21-4.89 L	14.3%	Severely reduced
Forced vital capacity (FVC)	1.80 L	3.74-5.75 L	37.9%	Severely reduced
FEV_1_/FVC ratio	0.63	0.70-0.94	76%	Indicative of obstructive pattern
Total lung capacity (TLC)	7.12 L	5.27-7.57 L	110.8%	Hyperinflation
Residual volume (RV)	5.62 L	0.86-2.21 L	366.4%	Significantly increased
Functional residual capacity (FRC)	5.62 L	2.24-4.05 L	139.0%	Increased
RV/TLC ratio	78.95%	14.73-32.69%	333.0%	Significantly increased, indicates air trapping
Transfer factor for carbon monoxide (TLCO)	4.22 mmol/min/kPa	8.78-13.42 mmol/min/kPa	38.0%	Severely reduced
Alveolar volume (VA)	2.19 L	6.27 L	35.0%	Severely reduced
Carbon monoxide transfer coefficient (KCO)	1.93 mmol/min/kPa/L	3.23-4.22 mmol/min/kPa/L	59.7%	Moderately reduced

**Figure 6:**
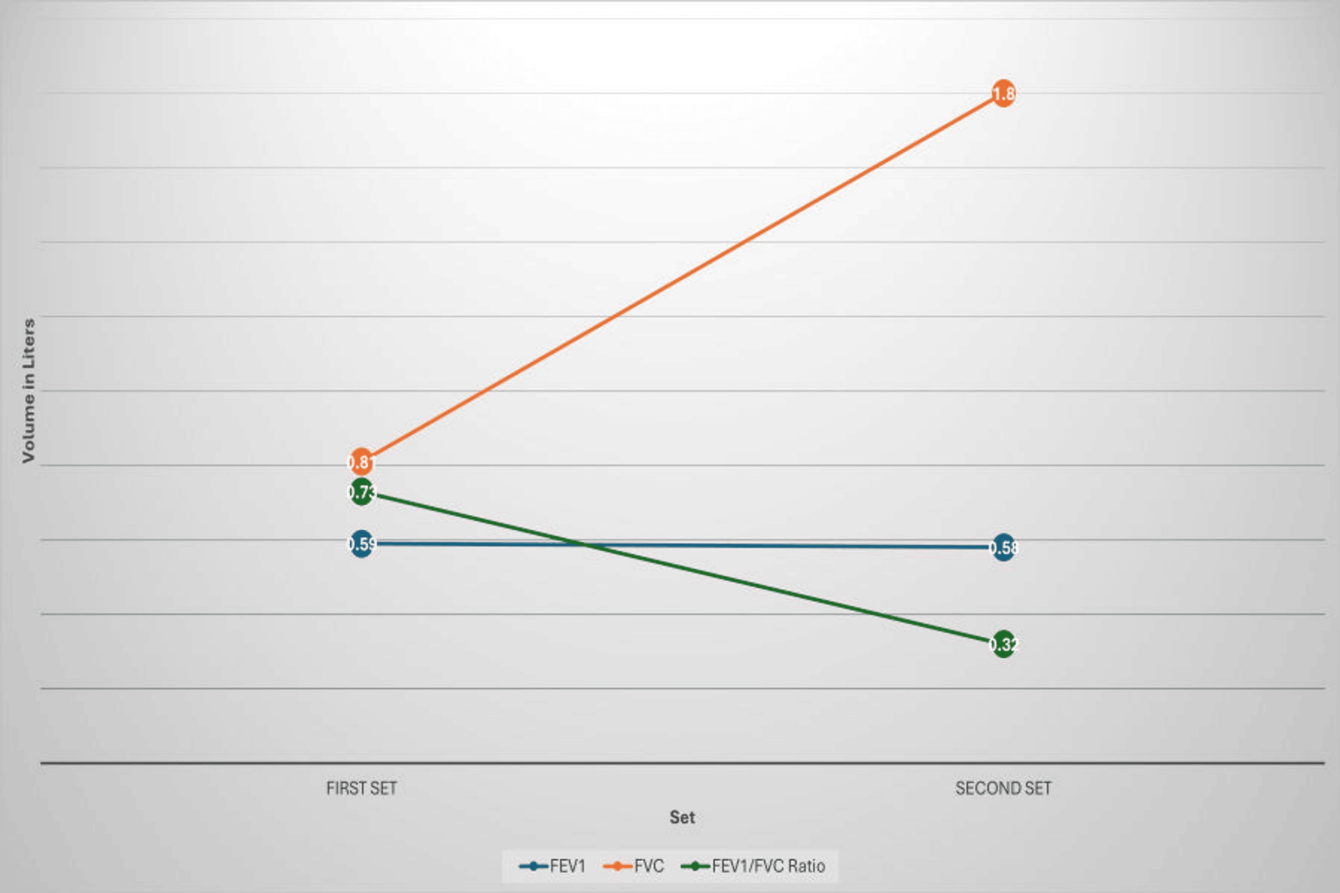
Spirometry trends in two sets of measurements: FEV₁, FVC, and FEV₁/FVC ratio

Lung function tests demonstrated severe obstruction, with a reduced FEV_1_ and FVC, showing slight improvement compared to the results from the index admission (Figure 6). Peak expiratory flow (PEF) remained reduced, and the FEV_1_/FVC ratio indicated an obstructive pattern. The tests also revealed hyperinflation and significant air trapping, with an elevated total lung capacity (TLC) and markedly high residual volume (RV) to TLC (RV/TLC) ratio. These findings are consistent with the diagnosis of severe obstructive lung disease, most likely due to post-SJS BO.

**Figure 7:**
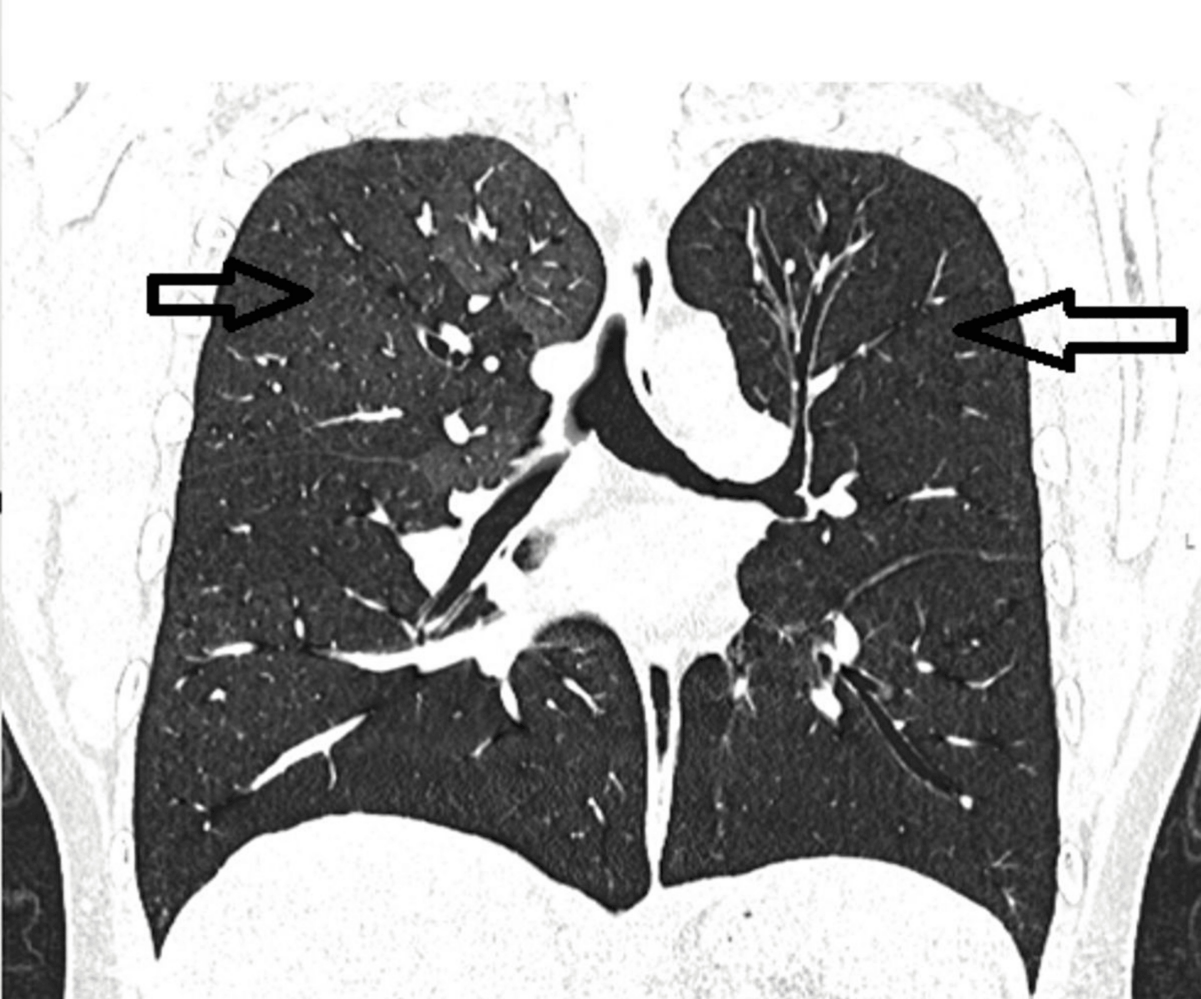
High-resolution computed tomography (HRCT) of the chest, coronal section, inspiratory phase

**Figure 8:**
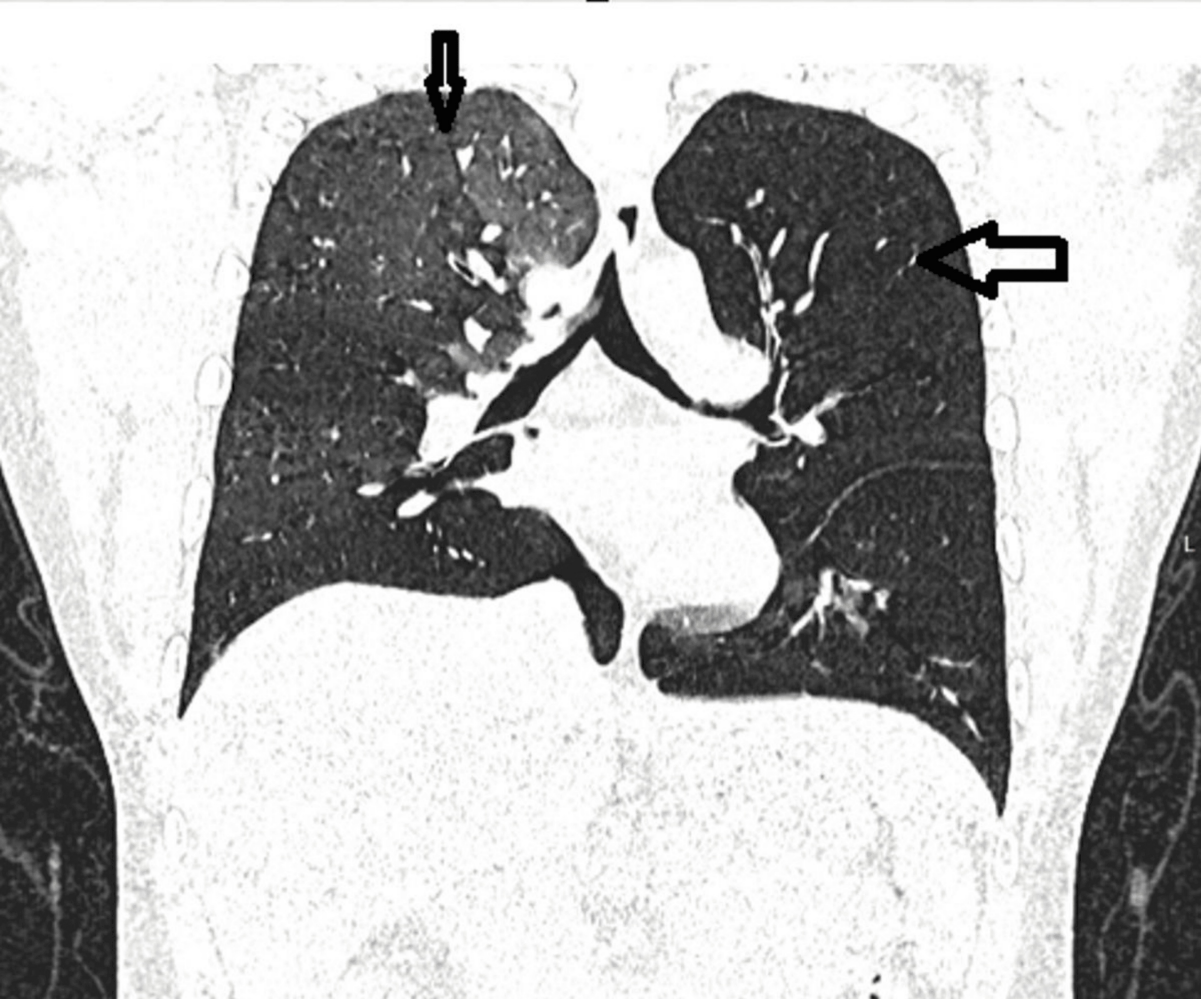
High-resolution computed tomography (HRCT) of the chest, coronal section, expiratory phase

The patient underwent a follow-up HRCT chest scan in both inspiratory and expiratory phases (Figure 7 and Figure 8), which revealed a mild improvement in air trapping in the right lung. However, persistent air trapping and hyperinflation were observed in the left lung.

Following thorough discussions with the lung transplantation multidisciplinary team at a tertiary hospital, the patient is currently undergoing assessment for lung transplantation because of the limited improvement in lung function test results and the generally poor prognosis of BO without transplantation [2].

## Discussion

SJS and toxic epidermal necrolysis (TEN) are severe, potentially life-threatening dermatologic emergencies characterised by extensive skin and mucosal necrosis. Although both share similar pathophysiological mechanisms, they differ primarily in the extent of body surface area (BSA) involvement. SJS involves less than 10% BSA, TEN involves more than 30% BSA, and SJS/TEN overlap affects between 10% and 30% BSA.

Pathogenesis

The primary cause of SJS/TEN is adverse drug reactions, most commonly triggered by medications, such as antiepileptics, sulfonamides, antibiotics, and allopurinol. Certain infections, such as those caused by *Mycoplasma pneumoniae*, may also cause SJS. Pathogenesis involves an immune-mediated response that causes widespread apoptosis of keratinocytes, which is driven by cytotoxic T-cells and natural killer cells. Soluble Fas ligand and granulysin are significant mediators of this response, leading to the hallmark epidermal necrolysis observed in SJS/TEN [3].

Complications associated with SJS and TEN are diverse, affecting multiple systems. Common complications include the following:

Ocular complications: Ocular damage is prevalent, with severe cases leading to conjunctival sloughing, symblepharon formation, and, in some cases, blindness. Early ophthalmologic intervention can mitigate long-term ocular sequelae [3].

Respiratory complications: SJS and TEN are reported to be associated with various chronic pulmonary complications, which include BO, bronchiectasis, chronic bronchitis, and other forms of obstructive and interstitial lung disease. Other less common manifestations include BO organising pneumonia (BOOP), obstructive pulmonary disease, and pneumopathy with pulmonary perfusion deficits. These findings highlight that the most frequent chronic pulmonary complications associated with SJS or TEN are chronic bronchitis/bronchiolitis with obstructive impairment, including BO, BOOP, bronchiectasis, and respiratory tract obstruction [4].

Gastrointestinal and renal complications: These include erosion of the gastrointestinal tract and renal failure, especially in severe TEN cases. Acute renal failure has been observed due to a high systemic inflammatory response and potential sepsis [3].

Infectious complications: Patients with BO are highly susceptible to secondary infections, a major cause of mortality. The disruption of the skin barrier combined with immunosuppressive treatments predisposes these patients to life-threatening infections.

SJS has an estimated incidence of 5.76 cases per million persons per year in the UK, while the US reports 9.2 cases per million for SJS and 1.6 per million for TEN. These conditions are more prevalent in females, with a female-to-male ratio of approximately 1.5:1. The Severity-of-Illness Score for Toxic Epidermal Necrolysis (SCORTEN) score is commonly used to assess SJS/TEN severity and to predict outcomes. Mortality rates vary by BSA involvement: SJS has a mortality rate of 4.8-9%, SJS/TEN overlap 19.4-29%, and TEN 14.8-48% in severe cases. Early identification and withdrawal of the causative agent are crucial for reducing mortality.

Management focuses on supportive care, usually requiring burn unit or intensive care unit (ICU) settings, with careful attention to fluid and electrolyte management, infection control, and wound care. Adjunctive treatments may include corticosteroids, intravenous immunoglobulin (IVIG), and cyclosporine, although there is no universal consensus on the most effective regimen. Cyclosporine, especially when combined with IVIG, has shown positive results in improving survival in patients with extensive BSA involvement [3].

BO, also known as obliterative bronchiolitis or constrictive bronchiolitis, is a chronic obstructive lung disease characterised by inflammation and fibrosis of bronchioles. This condition leads to narrowing and eventual obliteration of the bronchiolar lumen, causing airflow obstruction.

Causes

BO can arise from various conditions and is often associated with an immune response or direct injury to the bronchiolar epithelium. Common causes include the following.

Infectious Agents

Childhood respiratory infections include adenovirus, respiratory syncytial virus, measles virus, influenza virus, parainfluenza virus, and *Mycoplasma pneumoniae*.

Post-transplant Complications

Chronic rejection following lung and heart-lung transplantation, as well as chronic graft-versus-host disease (GVHD) after allogeneic hematopoietic stem cell transplantation.

Inhalational Injuries

Exposure to toxic fumes (e.g., ammonia, chlorine, and nitrogen dioxide).

Connective Tissue Disorders

It is particularly common in rheumatoid arthritis and occasionally seen with Sjögren’s syndrome, systemic lupus erythematosus, and scleroderma.

Medications

Use of drugs, such as D-penicillamine and certain chemotherapeutic agents.

Miscellaneous

SJS, paraneoplastic pemphigus, asthma, sarcoidosis.

Idiopathic

BO typically presents with symptoms, such as dyspnoea and wheezing, that do not respond to bronchodilators or corticosteroids, along with severe non-reversible obstructive pulmonary dysfunction and reduced diffusion capacity (diffusing capacity of the lungs for carbon monoxide (DLCO)). In the early stages, chest radiographs may appear normal or show signs of hyperinflation. Bronchoscopy findings can include central bronchiectasis and obstruction of peripheral bronchi.

HRCT is the preferred imaging modality for diagnosing BO. It commonly shows mosaic attenuation owing to heterogeneous ventilation, with air trapping seen on expiratory images. Other findings include bronchial wall thickening and bronchiectasis. These features are often sufficient for diagnosis, thus minimising the need for invasive biopsies. Although lung biopsy has historically been the diagnostic gold standard, the patchy distribution of BO lesions can lead to sampling errors, resulting in normal biopsy findings in up to one-third of the cases. HRCT findings are also essential to distinguish BO from other obstructive lung diseases [5-6].

Post-SJS BO was first identified in the 1980s by Reyes de la Rocha et al. [7]. In a literature review of 23 published case reports, the age of onset for BO following (SJS) or (TEN) ranged from five to 59 years, with 14 cases involving females [8]. Another review specifically examined paediatric patients and identified 36 cases in children aged five and 14 years [9]. In both reviews, BO onset occurred between five days and five months after the initial SJS/TEN episode. This aligns with our case, where respiratory symptoms indicative of BO began two weeks after the onset of SJS, supporting the timeline observed in previous studies.

BO was first observed in heart-lung transplant recipients in 1984, where it was termed "bronchiolitis obliterans syndrome" (BOS). BOS is defined by a persistent decline in FEV₁ to less than 80% of the predicted value, lasting for more than three weeks, due to progressive obliteration of the small airways. This condition reflects chronic rejection and immune-mediated damage to bronchioles, leading to a gradual decline in lung function and chronic respiratory impairment [10].

The exact mechanism of BO following SJS is not fully understood, but it is thought to involve immune complex deposition in the bronchiolar epithelium. This triggers an inflammatory response that causes mucosal damage and fibrosis, which leads to progressive obstruction of the bronchiolar lumen. Eosinophils and transforming growth factor (TGF)-β may contribute, particularly in the early stages, to the elevated IgE levels observed in this patient [11]. Post-infectious BO, which is a differential diagnosis for this patient, results from respiratory infections that cause epithelial injury, triggering inflammatory and fibrotic responses involving neutrophils, cluster of differentiation 8 (CD8+) T cells, T-helper 17 (Th17) cells, and profibrotic cytokines [12].

BO is characterised by extensive bronchiolar damage, fibrosis, and inflammation. Macroscopically, it presents with whitish nodules and dilated bronchioles. Histopathologically, BO exhibits fibrous obstruction of bronchioles, goblet cell hyperplasia, and proliferation of elastic fibres. Elevated TGF-β1 levels highlight the underlying state of chronic inflammation and fibrosis [11].

The prognosis for BO following SJS or TEN is generally poor, with patients often experiencing chronic respiratory symptoms, high morbidity, and limited functional improvement despite therapy [13]. Mortality rates are significant, particularly in severe cases and among paediatric patients, with some succumbing to the disease within a few years [9]. Additionally, complications, such as pneumothorax, are commonly reported, worsening clinical outcomes and increasing the risk of mortality [14]. Although lung transplantation has shown potential survival benefits in certain cases, its limited availability restricts its use as a treatment option, emphasising the need for close monitoring and further research into effective therapies.

Management

Azithromycin

Although specific studies on azithromycin use in post-SJS BO are lacking, evidence from case reports suggests its potential benefits [5]. Azithromycin improved BO syndrome (BOS) secondary to other causes, such as lung transplantation, with 35-40% of patients experiencing positive outcomes, including complete reversal of FEV1 decline in some cases [15].

FAM therapy, consisting of fluticasone (an inhaled corticosteroid), azithromycin (an antibiotic with anti-inflammatory properties), and montelukast (a leukotriene receptor antagonist), is used to treat BO syndrome (BOS), particularly following allogeneic hematopoietic cell transplantation.

Studies have demonstrated that FAM is well tolerated and can significantly reduce the rate of pulmonary function decline in the short-term. Patients have also reported improvements in their quality of life and reductions in systemic steroid use. However, FAM may not fully prevent BOS progression, underscoring the need for additional therapeutic options in severe cases [16].

Steroids

There is no clear consensus on steroid use in post-SJS BO; some reports suggest potential benefits [8], while others indicate a poor response [7]. In our patient, high-dose steroids during SJS treatment did not prevent the deterioration of lung function. Clinical improvement was observed only after starting FAM therapy and tapering off steroids, highlighting FAM’s potential efficacy of FAM in the management of his condition.

Azathioprine

In a reported case of post-SJS BO, azathioprine demonstrated good outcomes after one year of follow-up. Improvements were observed in clinical parameters, oxygenation levels, and PFTs, suggesting a potential benefit in managing this condition [17].

Rituximab

Multiple reports have documented the use of rituximab in BO secondary to pulmonary GVHD [18] and connective tissue disease-associated BO [19], showing improvements in PFTs in most patients and allowing for steroid reduction or complete weaning. This finding suggests the potential of rituximab as a steroid-sparing agent in these contexts. Although the pathogenesis of GVHD and connective tissue disease-associated BO differs from that of post-SJS BO, further studies are needed to clarify the role of rituximab in managing post-SJS BO.

Ruxolitinib

Ruxolitinib has proven to be an effective steroid-sparing agent in children with steroid-refractory or steroid-dependent BOS following allogeneic hematopoietic cell transplantation. However, further studies are needed to clarify its potential role in managing post-SJS BO [20].

Cyclophosphamide

Cyclophosphamide has been used as an adjunctive therapy in BO secondary to rheumatoid arthritis, with reports of rapid improvement following its administration. For patients with BO who do not respond adequately to corticosteroids, early therapeutic trials of cyclophosphamide may be considered a potential treatment option [21]. However, the role of cyclophosphamide in post-SJS BO remains unknown.

Lung Transplantation

A case report indicated that BO syndrome (BOS) induced by SJS/TEN may have favourable long-term outcomes after lung transplantation. However, additional prospective studies are required to validate this observation [22].

## Conclusions

Post-SJS BO is a severe chronic pulmonary complication with limited treatment options and a generally poor prognosis. This case underscores the importance of the early identification and individualised management of post-SJS BO. Although corticosteroids are commonly used in the early stages, their effectiveness remains uncertain, with patients often showing a minimal response to traditional asthma therapies. Our patient demonstrated clinical improvement only with the implementation of FAM therapy, suggesting its potential as a more beneficial early strategy for post-SJS BO patients compared to systemic steroids.

Lung transplantation may offer a viable option for patients with refractory or progressive disease, although its accessibility and risk of complications restrict its widespread application. Current literature suggests that long-term outcomes may improve with lung transplantation in select cases; however, further studies are essential to validate these findings and explore additional therapeutic avenues. This case contributes to the growing understanding of post-SJS BO and underscores the need for a multidisciplinary approach to optimise patient outcomes.
